# Neonatal lupus erythematosus: a cutaneous cases based update

**DOI:** 10.1186/s13052-015-0208-5

**Published:** 2016-01-07

**Authors:** Francesco Savino, Serena Viola, Valentina Tarasco, Emanuela Locatelli, Alberto Ricagni, Paola Coppo

**Affiliations:** Department of Pediatrics and Adolescence - Regina Margherita Children’s Hospital, Azienda Ospedaliera Città della Salute e della Scienza di Torino, University of Turin, Piazza Polonia, 94, Torino, 10126 Italy; Department of Surgery, Dermatology Unit, Regina Margherita Children’s Hospital, University of Turin, Turin, Italy

**Keywords:** Neonatal lupus erythematosus, Antinuclear antibodies, Skin lesions

## Abstract

**Background:**

Neonatal Lupus Erythematosus (NLE) is an uncommon autoimmune disease characterized by cutaneous, hepatic, hematological, neurological and cardiac involvement.

**Case presentation:**

Here we report four cases of cutaneous NLE which were referred to our department in the last 10 years and update literature. The newborns presented with different skin, clinical and laboratory features. This underlines the phenotypic variability of NLE. We investigated the passage of maternal antinuclear antibodies (ANA) and extractable nuclear antigen antibodies (ENA) - particularly anti-Ro/SSA, anti-La/SSB and anti-U1 ribonucleoprotein RNP - through the placenta. Despite the positive family background, cutaneous NLE and serological data improved in infants within 4 months without treatment.

**Conclusion:**

The evolution of cutaneous NLE may be the spontaneous regression of lesions within six months without progression to Systemic Lupus Erytehmatosus.

## Background

Neonatal lupus erythematous (NLE) is an uncommon autoimmune disease, first described by McCuiston and Schoch in 1954 [1]. It is caused by the passage of maternal antinuclear antibodies (ANA) and extractable nuclear antigen antibodies (ENA) through the placenta [2]. The most important ENA in the pathogenesis of disease are anti-Ro/SSA, anti-La/SSB and less frequently anti-U1 ribonucleoprotein RNP. At diagnosis, mothers are asymptomatic in 40 to 60 % of cases. Antinuclear antibody tests could be used as a screening test in mothers or patients suspected of having neonatal lupus erythematosus [3].

The most common manifestations are cutaneous lesions, hematological or hepatic abnormalities and congenital heart block [4, 5]. The cutaneous findings are variable and usually begin in the first weeks of life and improve within 4 -6 months.

Four infants presenting with different cutaneous features are described in this report, showing clinical and laboratory differences which underline the variability of this condition.

What is already known: Neonatal lupus erythematous is a rare neonatal disease.

Novel Insights: Despite the positive family background cutaneous and serological data resolution of skin lesions and serological data may occur in four months without treatment.

## Case presentation

### Case 1

**Fig. 1:**
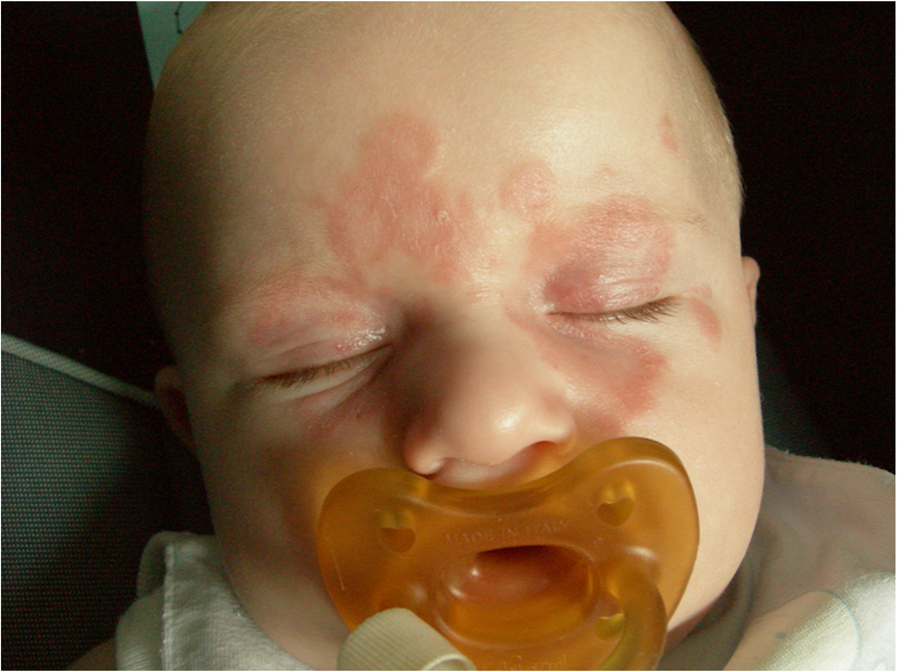
case 1: Newborn with erythematous eruption with annular patches and central regression, in particular over forehead and around lips

The infant was born via spontaneous delivery, after premature rupture of membranes. Two days after birth he was diagnosed with urinary tract infection and treated with antibiotics for 10 days. Then he was discharged, without any other problems. At 3 months of age, he started to present skin lesions on the face and upper limbs characterised by purpuric and erythematous features. Suspecting a cutaneous mycosis, he was initially but unsuccessfully treated with topic and systemic antifungal agents for two weeks. Then, he was referred to our Dermatologic Unit. At clinical examination, the infant presented erythematous eruption with annular patches and central regression, in particular over the forehead and around the lips (Fig. 1). No further alterations were observed in the clinical evaluation. Laboratory investigations at 3 months of age showed initial mild anemia and thrombocytopenia that then improved. IgA and IgM levels resulted quite low, reactive ANA (1 > 320) with a fine-speckled nuclear pattern, reactive anti-Ro/SSA (190 U/mL, normal value 0.00-10.00) and anti-La/SSB (220 U/mL, normal value 0.00-10.00). Urine tests, creatinine, C3 and C4 levels were normal. An electrocardiogram (ECG) did not reveal heart block or any other conduction defect. The mother’s laboratory investigation showed reactive ANA (1 > 640) with a fine-speckled nuclear pattern, reactive anti-Ro/SSA (>240 U/mL, normal value 0.00-10.00) and anti-La/SSB (> 320 U/mL, normal value 0.00-10.00). She (33 year- old) was diagnosed as suffering from asymptomatic Sjögren Syndrome. Laboratory tests of the newborn were negative. Family history, clinical and laboratory features were suggestive of NLE, misdiagnosed as cutaneous mycosis. The family was instructed to reduce sun exposure, use protective clothing and use sunscreen daily. Topic therapy with hydrocortisone was started and performed for three weeks, hematologic tests and laboratory tests were normal at 6 months of age.

### Case 2

**Fig. 2:**
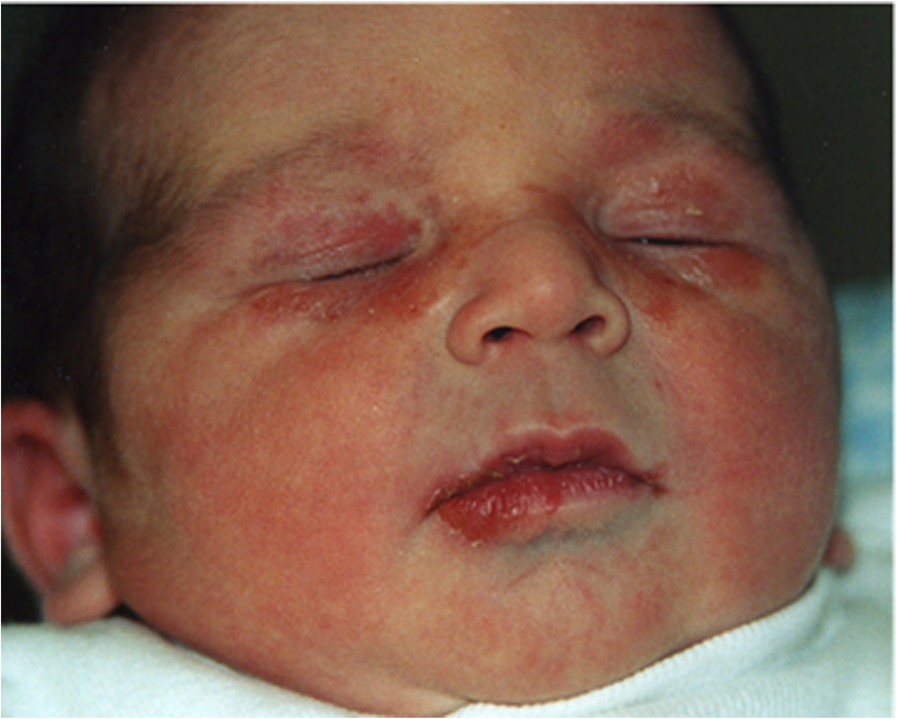
Case 2: Newborn with upper and lower eyelid angiomatous-like lesions featuring “eye-mask”

A full-term female was born to a 35-year-old from Perù woman affected by systemic lupus erythematosus (SLE). The serology tests of the newborn’s mother were positive for both anti-Ro/SSA (186 U/mL) and anti-La/SSB (205 U/mL); her anti-ds-DNA antibodies were negative, anti-CCP IgG and Rheumatoid factor were positive and C4 was reduced. The pregnancy was not complicated, all the ultrasound scan controls were normal and the fetus did not manifest any antenatal signs of congenital heart block. Two days after birth, the newborn was referred to our Department for the presence of upper and lower eyelid angiomatous-like lesions featuring “eye-mask” (Fig. 2). She presented unusual congenital erythematous lesions with very fine scale and central clearing on her face and a patch on her retroauricular region. No other lesions were noted. The clinical setting and the family history were suggestive for NLE, so laboratory evaluation was performed. It was significant for a high-titer ANA (>1/640) with a homogeneous immunofluorescent pattern. ENA count was elevated (> 32 ratio, normal value 0.00-1.00), with both anti- Ro/SSA (268 U/mL) and anti- La/SSB (320 U/mL); ENA Sm, ENA Sm/RNP, ENA Scl70 and ENA anti Jo1 were negative. Hemoglobin and platelet count were in the normal range. An ECG was normal. The diagnosis of NLE was confirmed and careful sun-protection of the skin was recommended. Because of the paucity of skin lesions, steroidal therapy was not started. Three months later the skin lesions improved and a reduction in ANA titer (1/320) was observed. Total ENA count was still elevated, but we noted a reduction in anti- Ro/SSA (193 U/mL). C3 and C4 were normal. At 7 months of age ANA titer was completely normalized and both anti-Ro/SSA and anti-La/SSB further decreased. The ECG remained normal. We followed up the patient annually for more than 4 years and she remained asymptomatic for SLE, with complete normalization of serology.

### Case 3

**Fig. 3:**
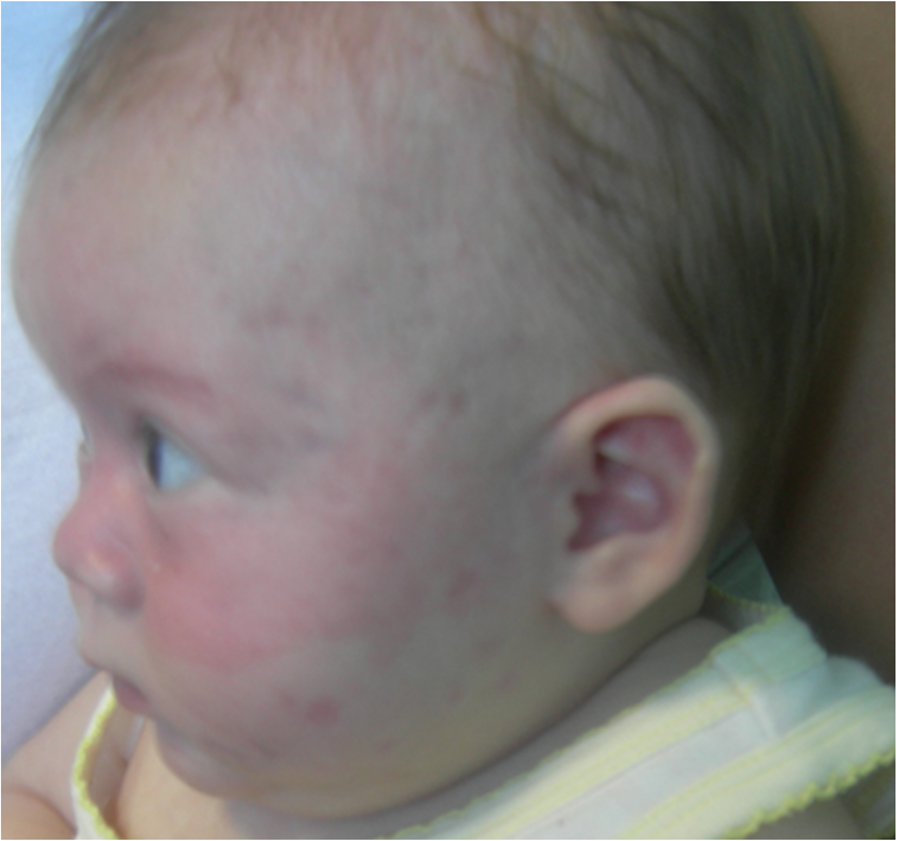
case 3: Newborn with widespread fine scale erythematous lesions, occurring on the face, in particular on lateral edges of eyes and involving cheeks

A 3-months old female infant presented cutaneous lesions a few days after birth. She was born at term, after an uncomplicated pregnancy and delivery. Personal and family histories were negative. Clinical examination, revealed widespread fine scale erythematous lesions, occurring on the face, in particular on the lateral edges of eyes and involving cheeks (Fig. 3). Slightly purplish atrophic areas were observed as spreading into the temple regions bilaterally. The exams showed positive ENA (3.8 Ratio) and anti-Ro/SSA (226 U/mL), with negative ANA, anti-La/SSB, anti-Sm, anti RNP, anti-Jo1, anti-Scl70 and anti CENP. Renal function tests, albumin, calcium, bilirubin, transaminases, erythrocyte sedimentation rate (ESR) and all other tests were normal. An ECG was normal. A diagnosis of NLE was made and the infant was treated with topic sunscreen. Follow up has not shown any complications and serology for ENA and antiRo/SSA was normal at 6 months of age. The 32 year old mother’s lab data were positive for both anti-Ro/SSA (191 U/mL), anti La/SSB (210 U/mL).

### Case 4

**Fig. 4:**
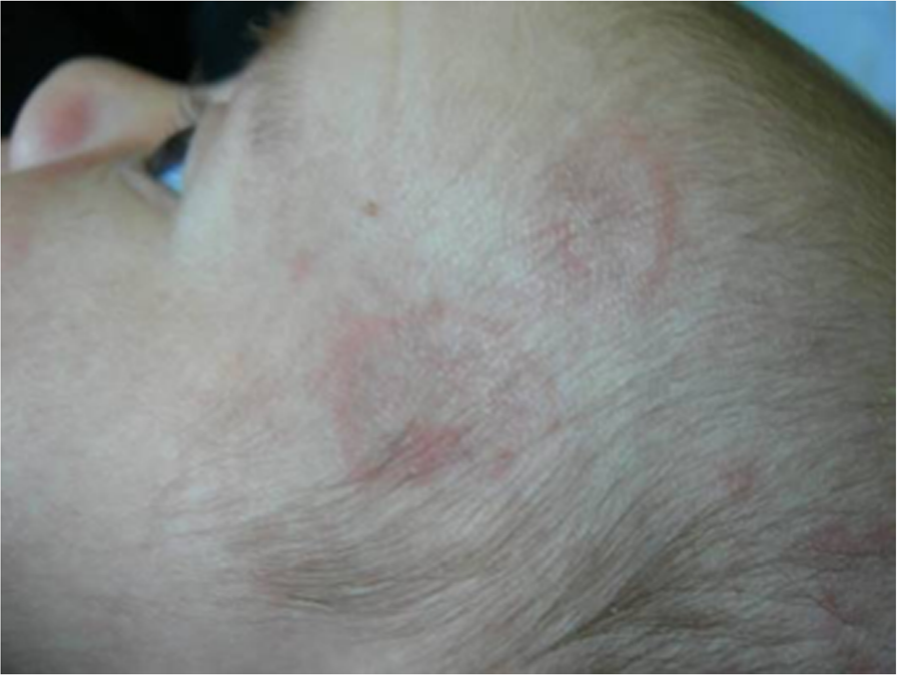
case 4: Newborn with annular erythematosus lesions, particularly located on the frontal-temporal surface, with a mildly atrophic central area

The infant was referred to our Dermatologic Unit for annular erythematosus lesions, located particularly on the frontal-temporal surface, with a mildly atrophic central area (Fig. 4). His mother (40 year-old) was affected by Sjögren Syndrome with positive Ro/SSA (212 U/mL), La/SSB (195 U/mL) and Rheumatoid factor. The newborn presented positive serology for ENA, in particular for Ro/SSA antibodies (> 47 U/mL), whereas La/SSB, Sm, RNP, Scl70, JO1 and CENP were negative. The ECG was normal. A clinical diagnosis of NLE was made, based on cutaneous features and mother’s history. Sun protection was proposed, without any pharmacological therapy. All the cutaneous lesions disappeared in seven months and serology was negative.

## Conclusion

Neonatal Lupus Erythematosus is a rare neonatal immune mediated disease. The true incidence is not yet defined, because of underdiagnosis and misdiagnosis; however, it is approximately 1:20000 live births and can affect all ethnic groups [3]. Females are affected twice as often as males [4]. In our Dermatologic Unit we visited approximately 15600 children from 2003 to 2013 and four of them were diagnosed with NLE. It is triggered by transplacental passage of maternal IgG against Ro/SSA, La/SSB and U1-RNP, after 16 weeks of gestational age. Anti-La/SSB antibodies influence the development of cutaneous NLE; instead anti-Ro/SSA antibodies are involved in the development of NLE with complete heart block. Other antibodies can be present, such as anti-calreticulin, anti-fodrinand antibodies against a 57 kDa protein and against a 75 kDa phosphoprotein [5]. Therefore, fetal genetic components may contribute to the pathogenesis of NLE or amplify the effect of the antibodies [6]. As the IgG are maternally derived, cutaneous forms of NLE are generally self-limiting in six-eight months [7].

We found positive maternal serologies for Ro/SSA and La/SSB in all the cases and one of them also presented a positivity of anti-CCP and Rheumatoid factor, with a reduction in C4.

About 50 % of women with circulating auto-antibodies who have children with NLE are asymptomatic, and some of them will develop some kind of rheumatologic disease, particularly Sjögren Syndrome, SLE and less often mixed connective tissue diseases [3].

In our case series, one mother was affected by Sjögren Syndrome, but she was asymptomatic and the diagnosis was performed after NLE identification in her son; the second one suffered from an already known SLE and the third one remained asymptomatic with anti-Ro/SSA; the fourth mother was affected by Sjogren Syndrome.

Typical manifestations of NLE include transient dermatitis, hepatic and hematologic abnormalities and congenital heart block (Table 1) [8]. Non cutaneous manifestations are described as quite frequent [2], but only one of our cases had hematologic anormalities. Nobody of them presented hepatic failure and only in one we reported a decrease in platelets count with spontaneous improvement. The most common extra-cutaneous manifestation is cardiac involvement, which occurs in 2 % of newborns whose mothers are positive for Ro/SSA or La/SSB [9]. The most serious complication is atrioventricular block, which can be diagnosed in utero with a routine ultrasound scan and is commonly referred to as congenital complete block [10]. In our cases all pregnancies were uncomplicated and all the ultrasound controls were normal without any antenatal signs of congenital heart block.

**Table 1:** NLE features

Cutaneous features	Transient dermatitis with characteristic rash periorbital lesions or angiomatous-like lesions
Hematological features	Hemolytic anemia, neutropenia, trombocytopenia
Liver features	Hepatic abnormalities such as cholestasis and cytolsis
Cardiac features	Congenital heart block, endocardial fibroelastosis and dilated cardiomyopathy
Neurological features	Subependymal pseudocysts and Subependymal hemorrhage
Histology	Basal cell vasculopathy and mononuclear cell infiltration
Laboratory tests	Anti-Ro/SSA or Anti La/SSB or U1-RNP antibodies in the mother and in the child

Skin lesions are similar to subacute cutaneous SLE and commonly consist of annular, erythematosus, scaly plaques. Teleangectasia may be present, as in our second case, and disordered angiogenesis can play a role in its etiology [4]. Cutaneous lesions are typically localized on the facial central areas and they can involve periocular, perioral, zygomatic and temporal areas. Other lesions can sometimes be found on the neck, scalps, arms.

Some criteria are reported in literature to define cutaneous NLE: characteristic lesions diagnosed within the first year, with a photographic documentation, histologic evidence of typical basal cell vasculopathy and mononuclear cell infiltration, and anti-Ro/SSA or La/SSB or U1-RNP antibodies in the mother or in the child. A median age of 6 weeks at diagnosis is described [4].

Only one of our cases presents early neonatal lesions, although early presentation is often reported in literature [2]. We did not perform the histological analysis, as it is usually unnecessary [5].

Some differential diagnoses should be taken into account considering age, clinical features and localization. *Seborrheic dermatitis* manifests rarely with round or annular pattern of lesions and the scaly phase is more evident and yellowish [11]. *Tinea capitis* is not usually diagnosed in newborns and the presence of another family or contact case is essential to justify the infection. Skin lesions have a centrifugal trend, with a more inflammatory nature [12]. *Eyelid teleangectasias* usually present as salmon patches. They are capillary malformations with whole skin over, not scaly, and they do not present a worsening evolution: within the first weeks of life they become clearer, they do not increase in number and they are rarely multiple and nummular [13]. *Erythema multiforme* usually presents annular lesions, but in the majority of cases is localized on extensor surface of arms and not on face; moreover it usually appears as a consequence of viral infection [14].

The typical evolution is the spontaneous regression of the lesions within four or six months. However, skin lesions with a rich inflammatory component, particularly on the fronto-temporal areas if misdiagnosed and not protected against the sun, can result in semi-permanent epidermic atrophy [4].

As concernes prognosis available data show that the majority of patients with NLE of the skin, liver, or blood have transient disease that spontaneously resolves after 4-6 months. Also central nervous system abnormalities are temporary such as Subependymal pseudocysts (SEPC) and subependymal hemorrhage (SEH) observed using Cerebral Ultrasound without any correlations to autoantibody levels [15]; whether some sequelae occur is still unclear [16].

NLE can have substantial associated morbidity and mortality if the heart is affected such as congenital heart block, endocardial fibroelastosis and dilated cardiomyopathy [17, 18].

The fourth of our patients presented fronto-temporal lesions with a mildly atrophic central area, but they did not result in permanent signs. In our patients, skin lesions improved in a few months and we observed progressive serological normalization.

No cases of SLE or renal lupus are reported in children who presented NLE (as we observed in our patients) [19–23].

When patients show skin lesions, exposure to direct sunlight should be avoided. Topical steroids sometimes reduce the evolution to atropy, whereas systemic steroids are not indicated [3].

In conclusion, cutaneous NLE is a rare neonatal disease with a variable phenotype that may regress by the age of 6 months. The diagnosis may be suggested by characteristic cutaneous lesions and different pathologies should be taken into account considering age, clinical features and localization. Our experience shows that the evolution of cutaneous NLE is the spontaneous regression of the lesions within six months without progression to SLE.

## Consent

Written informed consent was obtained from parents of the patients for publication of this Case report and any accompanying images. A copy of the written consent is available for review by the Editor-in-Chief of this journal.

## Abbreviations

NLE: Neonatal lupus erythematosus
ANA: Antinuclear antibodies
ENA: Extractable nuclear antigen antibodies
ECG: Electrocardiogram
SLE: Systemic lupus erythematosus
ESR: Erythrocyte sedimentation rate

## References

[1] McCuiston C, Schoch EP (1954). Possible discoid lupus erythematosus in newborn infant. Arch Dermatol.

[2] Diociaiuti A, Paone C, Giraldi L, Paradisi M, El Hachem M (2005). Congenital Lupus erythematosus: case report and review of the literature. Pediatr Dermatol.

[3] Wisuthsarewong W, Soongswang J, Chantor R (2011). Neonatal Lupus Erythematosus: clinical character, investigation, and outcome. Pediatr Dermatol.

[4] Neiman A, Lee L, Weston W, Buyon J (2000). Cutaneous manifestations of neonatal lupus without heart block: characteristics of mothers and children enrolled in a national registry. J Pediatr.

[5] Lee LA (2010). Cutaneous lupus in infancy and childhood. Lupus.

[6] Lee LA (2005). Transient autoimmunity related to maternal autoantibodies: neonatal lupus. Autoimmun Rev.

[7] Silverman E, Jaeggi E (2010). Non-cardiac manifestations of neonatal lupus erythematosus. Scand J immunol.

[8] Inzinger M, Salmhofer W, Binder B (2012). Neonatal Lupus Erythematosus and its clinical variability. J Dtsch Dermatol Ges.

[9] Marker-Hermann E, Fisher-Betz R (2010). Rheumatic disease and pregnancy. Curr Opin Obstet Gynecol.

[10] Buyon JP (1996). Neonatal Lupus: bedside to bench and back. Scand Rheumatol.

[11] Gupta AK, Bluhm R, Cooper EA, Summerbell RC, Batra R (2003). Seborrheic dermatitis. Dermatol Clin.

[12] Möhrenschlager M, Seidl HP, Ring J, Abeck D (2005). Pediatric tinea capitis: recognition and management. Am J Clin Dermatol.

[13] Leung AK, Telmesani AM (1989). Salmon patches in Caucasian children. Pediatr Dermatol.

[14] Sokumbi O, Wetter DA (2012). Clinical features, diagnosis, and treatment of erythema multiforme: a review for the practicing dermatologist. Int J Dermatol.

[15] Zuppa AA, Gallini F, De Luca D, Luciano R, Frezza S, de Turris PL (2004). Cerebral ultrasound findings in neonatal lupus syndrome. Biol Neonate.

[16] Chen CC, Lin KL, Chen CL, Wong AM, Huang JL (2013). Central nervous system manifestations of neonatal lupus: a systematic review. Lupus.

[17] Levesque K, Morel N, Maltret A, Baron G, Masseau A, Orquevaux P (2015). Description of 214 cases of autoimmune congenital heart block: Results of the“Lupus néonatal” group. Autoimmun Rev.

[18] Guettrot-Imbert G, Cohen L, Fermont L, Villain E, Francès C, Thiebaugeorges O (2011). A new presentation of neonatal lupus: 5 cases of isolated mild endocardial fibroelastosis associated with maternal Anti-SSA/Ro and Anti-SSB/La antibodies. J Rheumatol.

[19] Buyon JP, Clancy RM, Friedman DM (2009). Cardiac manifestations of neonatal lupus erythematosus: guidelines to management, integrating clues from the bench and bedside. Nat Clin Pract Rheumatol.

[20] Zuppa AA, Fracchiolla A, Cota F, Gallini F, Savarese I, D'Andrea V (2008). Infants born to mothers with anti-SSA/Ro autoantibodies: neonatal outcome and follow-up. Clin Pediatr (Phila).

[21] Singalavanija S, Limpongsanurak W, Aoongern S (2014). Neonatal lupus erythematosus: a 20-year retrospective study. J Med Assoc Thai.

[22] Mina R, Brunner HI (2013). Update on differences between childhood-onset and adult-onset systemic lupus erythematosus. Arthritis Res Ther.

[23] Li YQ, Wang Q, Luo Y, Zhao Y (2015). Neonatal lupus erythematosus: a review of 123 cases in China. Int J Rheum Dis.

